# Supplementary material for: The Hedgehog-GLI Pathway Regulates MEK5-ERK5 Expression and Activation in Melanoma Cells
Source: Int J Mol Sci. 2021 Oct 19;22(20):11259. doi: 10.3390/ijms222011259 (PMC8538987; doi:10.3390/ijms222011259)
Supplement: Supplementary file 1 [file ijms-22-11259-s001.zip › ijms-1396694-supplementary.pdf]

SUPPLEMENTARY MATERIALS

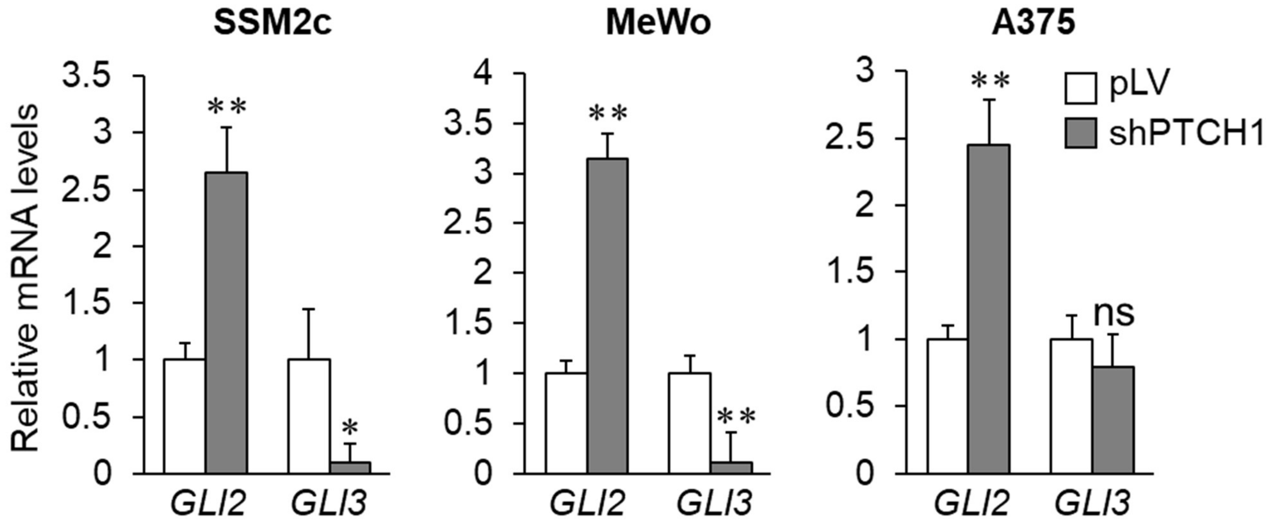

Supplementary Figure S1. Melanoma cells were lysed 5 days after transduction with control/empty lentiviral vectors (pLV) or lentiviral vectors carrying PTCH1-specific shRNA (shPTCH1), and *GLI2* and *GLI3* mRNA levels determined by Q-PCR. Data shown are mean ± SD from three independent experiments. *p <0.05, **p = 0.01 and ns = not significant as determined by Student's t-test.

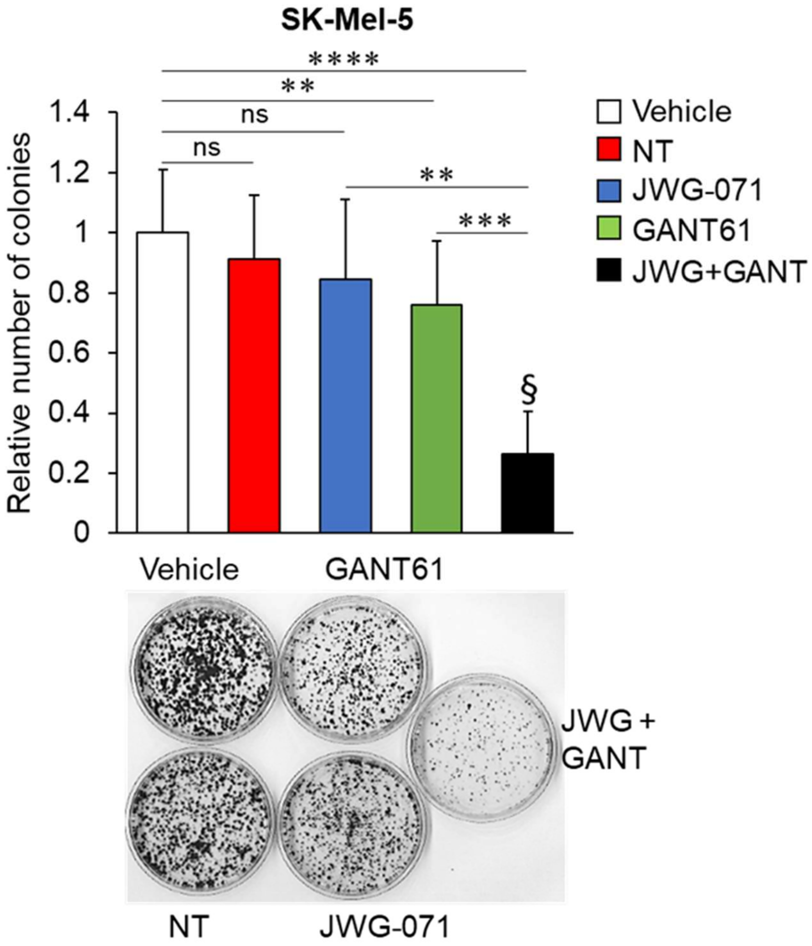

Supplementary Figure S2. Combined pharmacological inhibition of HH-GLI and ERK5 pathway synergistically reduces SK-Mel-5 cell proliferation. For colony formation assays, 3500 SK-Mel-5 cells were seeded in p60 dishes and treated with DMSO (Vehicle), JWG-071 (1 μM), GANT61 (1 μM), alone or in combination (JWG+GANT). Colonies (with more than 50 cells, i.e., 8 cell diameter) were counted following crystal violet staining after 14 days. Histograms represent mean ± SD from three independent experiments. Representative images of plates are shown. $^{**}p < 0.01$, *** $p < 0.001$, **** $p < 0.0001$ and ns = not significant as determined by Student's t-test. $^{\$}$Bliss independence score (>0) indicates synergistic effects over single treatments.

Supplementary Table S1. Genetic alterations of melanoma cell lines used in this study.

**Cell line**	**Alterations**
A375	BRAFV600E
SK-Mel-5	BRAFV600E
MeWo	NF1, p53, CDKN2A trunc/indel
SSM2c	wtBRAF / wtNRAS

Supplementary Table S2. List of the antibodies used and their application.

Protein	Use	Source	Notes	Cat. No.	Company
ERK5	WB	Rabbit polyclonal		#3372	Cell Signaling Technology, Danvers, MA, USA
pERK5-T218/Y220	WB	Rabbit polyclonal		#3371	Cell Signaling Technology, Danvers, MA, USA
MEK5	WB	Goat polyclonal	C-20	sc-1287	Santa Cruz Biotechnology, Santa Cruz, CA, USA
pMEK5-S311/T315	WB	Rabbit polyclonal		sc-135702	Santa Cruz Biotechnology, Santa Cruz, CA, USA
GLI1	WB/ChIP	Mouse monoclonal	L42B10	#2643	Cell Signaling Technology, Danvers, MA, USA
GLI2	WB	Mouse polyclonal		AF3635	R&D Systems, Minneapolis, MN, USA
GLI3	WB	Mouse polyclonal		AF3690	R&D Systems, Minneapolis, MN, USA
E2F1	WB	Rabbit polyclonal		#3742	Cell Signaling Technology, Danvers, MA, USA
RNApol II	ChIP	Mouse monoclonal	4H8	ab5408	Abcam, Cambridge, UK
IgG	ChIP	Rabbit anti-mouse		M7023	Sigma-Aldrich St. Louis, MO, USA
Actin	WB	Mouse monoclonal	AC-15	A1978	Sigma-Aldrich St. Louis, MO, USA
Vinculin	WB	Mouse monoclonal		V9131	Sigma-Aldrich St. Louis, MO, USA
IRDye 800CW	WB	Goat anti-rabbit		926-32211	LI-COR Biosciences, Lincoln, NE, USA
IRDye 800CW	WB	Goat anti-mouse		926-32210	LI-COR Biosciences, Lincoln, NE, USA
IRDye 800CW	WB	Donkey anti-goat		926-32214	LI-COR Biosciences, Lincoln, NE, USA
IRDye 680RD	WB	Goat anti-rabbit		926-68071	LI-COR Biosciences, Lincoln, NE, USA
IRDye 680RD	WB	Goat anti-mouse		926-68070	LI-COR Biosciences, Lincoln, NE, USA
IRDye 680RD	WB	Donkey anti-goat		926-68074	LI-COR Biosciences, Lincoln, NE, USA

Supplementary Table S3. List and sequences of the shRNA used in the present study.

Gene	Clone ID	shRNA	TRC Number	Sense sequence 5' to 3'
none	none	shNT	TRCN0000023236	CCGGCGACAATATCATCGCCATCAACTCGAGTTGATGGCGATGATATTGTCGTTTTT
MAPK7	NM_139032	shERK5-1	TRCN0000010262	CCGGGCTGCCCTGCTCAAGTCTTTGCTCGAGCAAAGACTTGAGCAGGGCAGCTTTTT
MAPK7	NM_139032	shERK5-2	TRCN0000010275	CCGGGCCAAGTACCATGATCCTGATCTCGAGATCAGGATCATGGTACTTGGCTTTTT
GLI1		shGLI1		CCTGATTATCTTCCTTCAGAA
PTCH1		shPTCH1		GCACTATGCTCCTTTCCTC

Supplementary Table S4. List and sequences of the primers used in the present study.

**List of primers used for Q-PCR**		
**Gene**		**Primer sequence (5' to 3')**
GLI1	Forward	CCCAGTACATGCTGGTGGTT
	Reverse	GCTTTACTGCAGCCCTCGT
GLI2	Forward	CACCGCTGCTCAAAGAGAA
	Reverse	TCTCCACGCCACTGTCATT
GLI3	Forward	CGAACAGATGTGAGCGAGAA
	Reverse	TTGATCAATGAGGCCCTCTC
MAPK7	Forward	TGCCCCACCAAAGAAAGATG
	Reverse	AAGACTTGAGCAGGGCAGCTT
MAP2K5	Forward	CCGTTCATCGTGCAGTTCAA
	Reverse	CCCGGCACACCCACAT
GAPDH	Forward	AACAGCCTCAAGATCATCAGCAA
	Reverse	CAGTCTGGGTGGCAGTGAT
18srRNA	Forward	CGGTACCACATCCAAGGAA
	Reverse	GCTGGAATTACCGCGGCT
**List of primers used for ChIP Assay**		
**Gene**		**Primer sequence (5' to 3')**
MAPK7 promoter		
1.1	Forward	CAGATAGGCGTGGGTTCAAT
	Reverse	TGGAGTTACTGTCCCGCGTT
2.1	Forward	TTGGGACCATAGAGGACTGC
	Reverse	TGCCCCTGAGGCCAGCCTAT
3.1	Forward	CGGTGCTCCATAAACATTTG
	Reverse	GCGGTTTCCTGGCTTCTAGG
3.2	Forward	ACGGTAGGTGGTCCTCTCCT
	Reverse	GATCTCGTACTCGTCGCCCA
4.1	Forward	ATGGGACATGGTTTGAGAGC
	Reverse	GCCACTCTCCCAGCTGCAGG
4.2	Forward	GTCACAGGGCTGGGATTAGA
	Reverse	GTCCCCATGTCCCTACCCCC
